# Machine Learning Augmented Echocardiography for Diastolic Function Assessment

**DOI:** 10.3389/fcvm.2021.711611

**Published:** 2021-08-04

**Authors:** Andrew J. Fletcher, Winok Lapidaire, Paul Leeson

**Affiliations:** ^1^Oxford Cardiovascular Clinical Research Facility, Division of Cardiovascular Medicine, Radcliffe Department of Medicine, University of Oxford, Oxford, United Kingdom; ^2^Department of Cardiac Physiology, Royal Papworth Hospital National Health Service Foundation Trust, Cambridge, United Kingdom

**Keywords:** artificial inteligence, echocardiogaphy, diastolic dysfunction, machine learning, heart failure preserved ejection fraction

## Abstract

Cardiac diastolic dysfunction is prevalent and is a diagnostic criterion for heart failure with preserved ejection fraction—a burgeoning global health issue. As gold-standard invasive haemodynamic assessment of diastolic function is not routinely performed, clinical guidelines advise using echocardiography measures to determine the grade of diastolic function. However, the current process has suboptimal accuracy, regular indeterminate classifications and is susceptible to confounding from comorbidities. Advances in artificial intelligence in recent years have created revolutionary ways to evaluate and integrate large quantities of cardiology data. Imaging is an area of particular strength for the sub-field of machine-learning, with evidence that trained algorithms can accurately discern cardiac structures, reliably estimate chamber volumes, and output systolic function metrics from echocardiographic images. In this review, we present the emerging field of machine-learning based echocardiographic diastolic function assessment. We summarise how machine-learning has made use of diastolic parameters to accurately differentiate pathology, to identify novel phenotypes within diastolic disease, and to grade diastolic function. Perspectives are given about how these innovations could be used to augment clinical practice, whilst areas for future investigation are identified.

## Introduction—Diastolic Assessment in Clinical Practice

Left-sided cardiac diastolic dysfunction can lead to patients developing debilitating symptoms such as dyspnoea and fatigue, as well as conferring worse survival and increased morbidity (1–3). Prevalence estimates of diastolic dysfunction vary widely depending upon the population studied and the definition used, but a recent review of community studies suggests it is in the range of 20-30% in the general population (4). In studies of thousands of patients in clinical settings, prevalences of 5.0% (5) to 9.2% (3) have been reported when accompanied by a normal left ventricular (LV) ejection fraction (EF) > 50%. Diastolic dysfunction is a criterion for the diagnosis of heart failure (HF) with preserved EF (HFpEF) (6), which represents about a third of all hospitalised heart failure in the United Kingdom (7) and about half of community heart failure in North America (8).

The gold standard for assessing diastolic function is invasive pressure-volume loop analysis, which directly measures ventricular compliance and relaxation, but is seldomly performed clinically as the process and analyses are technically challenging. The next best assessment technique is direct measurement of cardiac pressures with catheterisation, because diastolic pressures rise with advanced dysfunction. Cardiac catherisation can either be performed via left heart catheterisation (LHC), where an end diastolic pressure (LV-EDP) or pre-A wave pressure are the common benchmarks, or by right heart catheterisation (RHC), where a pulmonary capillary wedge pressure (PCWP) is recorded which approximates the LA pressure from across the pulmonary capillary bed. These invasive procedures are also not routinely performed in most patient cohorts as they are resource intensive whilst exposing the patient to radiation and possible discomfort. Therefore, echocardiography is often preferred for diastolic function assessment.

Echocardiography is the primary imaging tool used for assessing diastology and HFpEF in routine clinical practice because it is non-invasive and widely available. There are over 20 different variables of diastolic relevance that can be measured using routine transthoracic echocardiography (9), as well as variables emerging from the research domain such as speckle-tracking strain parameters (10). Unfortunately, no single echocardiographic parameter adequately captures the complexity of diastolic function, given the different structural and functional changes which can manifest at different time points during the cardiac cycle. Routine variables, commonly derived from pulse-wave Doppler and tissue Doppler techniques, at best modestly correlate with invasively measured diastolic pressures (11–13).

Diastolic function is therefore classified echocardiographically by combining multiple parameters. The most widely adopted method for this comes from the American Society of Echocardiography and European Association of Cardiovascular Imaging (ASE/EACVI) guideline (9). The method uses a series of decision steps in the form of two algorithms, one screening for the presence of diastolic dysfunction and the other to grade diastolic dysfunction if it is found to exist (or is assumed to exist based upon defined clinical and structural observations), however there are a number of caveats which complicate matters. Classification outcomes are either that the guideline cannot be applied due to insufficient requisite information, that the diastolic function is indeterminate or normal, or that diastolic function is graded as mild, moderate, or severely impaired. Filling-pressure, which refers to left atrial (LA) and/or LV diastolic pressures, is often dichotomously described as “normal” or “raised.” Moderate and severe ASE/EACVI guideline graded diastolic dysfunction correspond to raised filling-pressures (9).

## Limitations of Routine Diastolic Assessment

There are a number of barriers to widespread and robust diastolic evaluation with echocardiography, particularly with following guideline suggestions, which include: accuracy uncertainties, unclassifiable and indeterminate situations, and confounding from comorbidities. These introduce clinical uncertainty, which can lead to inappropriate treatment decisions, and are explored in more detail below.

### Clinical Guideline Accuracy

Dual echocardiographic and invasive-catheterisation validation studies show suboptimal accuracy of the current ASE/EACVI guideline method to identify patients with raised filling-pressures. Sato et al. demonstrated that guideline classified moderate or severe diastolic dysfunction predicted raised invasively measured filling-pressures with an accuracy of 66% in an all-comers clinical population receiving echocardiography and LHC within 24 h (1). Lancellotti et al. found an accuracy of 56% of the guideline for predicting raised filling-pressures in patients receiving LHC for known or suspected coronary artery disease (14). Balaney et al. reported an accuracy of 68% in patients attending for LHC for a variety of clinical indications (15). Andersen et al. demonstrated accuracy of 87% in patients having either LHC or RHC for any valid clinical reason in a multi-institutional study (16).

It is worth noting that all of these studies excluded patients with confounding factors before recruitment or analysis, so they may not represent “real-world” accuracies in all-comer populations. Unfortunately, varying methodologies of researchers also limits our ability to compare these results. For the invasive validation studies above, different definitions of raised filling-pressures were used [LV-EDP > 14 mmHg (14), LV-EDP > 15 mmHg (17), LV-EDP > 16 mmHg (1), pre-A pressure > 12 mmHg (15, 16), PCWP > 12 mmHg (16), and PCWP > 15 mmHg (17)]. Furthermore, whilst the ASE/EACVI guideline recommends using an average of medial and lateral mitral annular e′ values to calculate an average E/e′ ratio in the majority of pathologies (9), some institutions have historically only acquired one or the other, or only report one in publications, again limiting clinical applicability of results (18, 19).

### Unclassifiable and Indeterminate Diastolic Grading

Unclassifiable diastolic function can arise from key parameters, needed to follow the guideline decision steps, being missing. A frequent cause of this is a suboptimal acoustic window which precludes measurements. One author reported unclassifiable diastolic function in 8% of consecutive echocardiograms due to poor image quality and missing data (20), whilst another study found this in 22% of scans (3), showing that this situation occurs regularly.

Indeterminate diastolic grading also creates uncertainty and may result in additional resource intensive or higher risk investigations, like exercise echocardiography or cardiac catheterisation. In the guideline approach, it occurs because parameters required in the decision-steps are contradictory/inconclusive. This too is frequent—a report from the National Echocardiography Database of Australian (3) found 27% of 344,646 scans were labelled as indeterminate, whilst a study of consecutive Canadian tertiary centre echocardiograms found 36% indeterminate (5).

Further real-world clinical data from Europe (1,000 individuals), Britain (189 individuals), Asia (57,630 individuals), America (866 individuals), and Canada (71,727 individuals), show that even after excluding patients with diastology-confounding factors, between 11 and 22% of scans are labelled indeterminate (5, 18, 19, 21, 22). A pertinent limitation of the literature base collectively is that a breakdown of reasons for indeterminate grading is often not presented.

For data acquired under stricter research study protocols or during dual invasive validation studies, the indeterminate proportion is not too dissimilar at 7-24% (1, 14–16, 23, 24). However, in the setting of pulmonary hypertension, indeterminate classification may be as high as 53% (17) suggesting that confounding factors may magnify the indeterminate issue.

### Factors Confounding Diastolic Assessment

Pulmonary hypertension (PH), arrhythmias, tachycardia, and valvular pathologies can all complicate the assessment of diastolic function by confounding associations between individual diastolic parameters and filling-pressures, thus reducing diagnostic accuracy and sometimes even precluding the measurement of parameters altogether. Such situations are common and were found to occur in 52% of consecutive echocardiograms (20).

Atrial fibrillation not only causes technical problems concerning parameter measurement, due to variability in cardiac cycle length and potentially misleading LA dilation, but it also prevents any meaningful late-diastolic atrial pumping of blood into the LV. This removes key diastolic parameters like the E/A ratio. Between 48 and 57% of HFpEF patients have confounding atrial fibrillation (25, 26), highlighting the pressing need to overcome this obstacle. The routine diastolic parameter E/e' has suboptimal association with invasively measured filling-pressures in patients with atrial fibrillation (27), whilst a range of common diastolic parameters are known to be altered in atrial fibrillation when compared in the same patients to sinus rhythm (28), showing how difficult assessment is in the presence of arrhythmias.

Pre-capillary PH, that is PH not of a left-sided aetiology, results in left heart preload reduction, which creates a disconnect between the intrinsic left-sided diastolic state, its filling pressures and hence its echocardiographic parameters. It is not always clear at the time of echocardiography whether the PH aetiology is pre-capillary, post-capillary, or mixed, and hence diastolic assessment is often confounded. This situation may arise regularly given that PH occurs in 50-80% of HFpEF patients (29, 30). Leung et al. investigated patients referred for suspected PH with echocardiography and both LHC/RHC (17). The ASE/EACVI algorithm accuracy for identifying raised or normal filling-pressures was only 29 and 23%, respectively, although it must be noted that the guideline recommends emphasis of different parameters in situations such as PH, which are not applied in the same algorithmic way.

Left sided valvular disease is also known to confound traditional diastolic assessment due to variable influences upon individual parameters. Mitral stenosis and mitral annular calcification both reduce the e' velocity and uncouple the measure (and therefore E/e') from the underlying diastolic state and filling-pressure (31, 32). Significant mitral regurgitation raises the e' velocity, again uncoupling it from the intrinsic state and rendering it unreliable (31). Analysis of 161,468 echocardiograms excluded from diastolic classification due to confounding factors showed that mild or more mitral stenosis was present in 1.6% of these scans, moderate or greater mitral annular calcification in 1.2% and more than moderate mitral regurgitation in 3.3% (33). Given the huge quantity of echocardiograms performed annually globally, this represents confounding of a significant number of scans.

Confounding factors tend to be become more prevalent as diastolic function deteriorates and often coexist with HFpEF. For example, atrial fibrillation may develop within 4 years in a third of HFpEF patients who originally present in sinus rhythm (34). Echocardiographers are hence in need of techniques to better assess diastolic function in the presence of confounding factors, and more robust tools which reduce indeterminate and unclassifiable situations.

## Machine Learning Application to Diastolic Assessment

### Machine-Learning in Echocardiography

Machine-learning (ML), a domain of artificial intelligence born from advanced computer science, mathematical and statistical techniques, holds huge potential for improving echocardiographic analysis in terms of streamlining workflow, automating feature quantification and accurately identifying pathology (35). The term “supervised ML” refers to algorithms that are trained using data labelled with an important feature, outcome, or diagnosis. Supervised ML aims to perform a task which could be of a regression type, such as predicting an exact value of left atrial pressure, but in cardiology is often a classification type, for example to state whether a particular disease is present or not. Support vector machines, random forests, and artificial neural networks (deep-learning) are examples of supervised ML, where heart-failure hospitalisation or invasively measured left heart pressures could be suitable training labels.

Unsupervised ML refers to analyses which learn from unlabelled input data to perform the required task. Cluster analysis is such an algorithm, which for example can group patients with similar echocardiographic variable values to create novel stages of a disease process or can unearth homogeneous subgroups within a larger heterogeneous cohort. Another class of unsupervised ML is dimensionality reduction, where the input data is projected onto a lesser number of new variables, thus reducing complexity, increasing interpretability and visualisation, and making the dataset better primed for other ML techniques. Principle component analysis is an example of this, where original variables are mapped to a smaller number of new “principal component” variables, which retain as much of the original data variance as possible.

**Figure 1:**
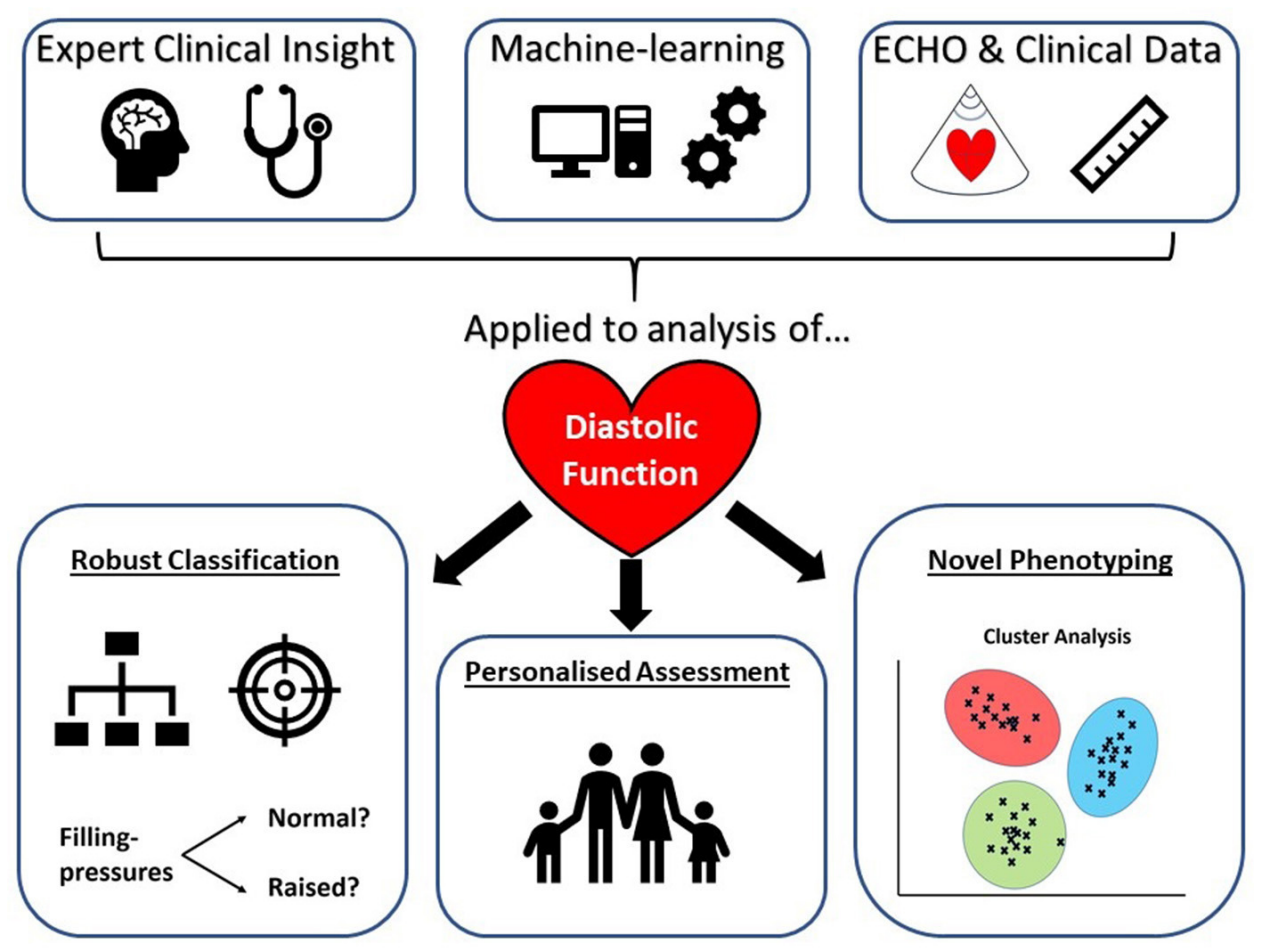
Machine learning augmented diastolic function assessment with echocardiography.

ML is thus a powerful tool able to process large high-dimensional datasets, such as those obtained with genomics, metabolomics, and imaging. It can learn not only from the acquired variables presented to it, but also by discerning novel features and latent data relationships (35, 36). Echocardiographic data, with its many parameters of diastolic function, combined with detailed clinical and demographic features is therefore well-suited as training material for ML models which could augment traditional diastolic assessment techniques (Figure 1). ML is particularly well-suited to detect and describe non-linear relationships (37), which is pertinent to diastolic assessment where, for example, the E/A ratio has a *U*-shaped relationship with dysfunction.

Much of the application of ML to echocardiography thus far has concerned automation and accuracy improvement of tasks such as image structure segmentation, left-sided chamber size calculation and estimation of systolic function metrics like ejection fraction (38–40). Prediction of the development of relevant pathology from images, such as clinically significant coronary artery disease, is also a focus of efforts given the prognostic implications (41). Attention is now increasingly being afforded to diastolic function, given the aforementioned challenges of contemporary assessment methodology, diastolic dysfunction prevalence, and suitability of echocardiographic data for training ML models.

### ML Integration of Diastolic Parameters

There are various applications of ML to echocardiographic diastolic function assessment—the key illustrative studies of which are summarised in Table 1. The first concerns the integration of diastolic parameters, whether collectively or alongside non-diastolic and/or non-echocardiographic variables, with ML to assist with disease diagnosis. Hubert et al. (42) report a new method for assessing diastolic function using strain-volume loops (SVL) derived from speckle-tracking strain imaging. SVL area differentiated between amyloidosis and HFpEF with an area under the receiver operator curve (AUC) of 0.76. However, when supervised linear discriminant analysis ML was applied to integrate the SVL with routine diastolic related echocardiographic parameters, the AUC increased to 0.91, showing the added value of individual diastolic variables.

**Table 1:** Summary of key publications demonstrating application of machine-learning to the echocardiographic assessment of diastolic function.

**References**	**Application category**	**ML technique(s)**	**Training data types**	**Key finding(s)**
Choi et al., (44)	Integration of diastolic parameters	Five techniques: “classification and regression tree” performed best.	1. Echocardiographic routine variables 2. Electrocardiogram variables 3. Clinical/haematological variables	When compared to physician diagnosis, ML diagnosed HFpEF with 99.6% concordance
Sanchez-Martinez et al., (36)	Novel diastolic variable discovery	Unsupervised agglomerative hierarchical clustering	1. Exercise echocardiography tissue-doppler variables	Premature fusion of early and late diastolic waves, increased variability in the onset of atrial contraction (a′ wave) and a blunted response in atrial velocities during exercise were novel diastolic variables for assessing HFpEF with ML.
Segar et al., (49)	Phenotyping, prognostication	Unsupervised penalized finite mixture model-based clustering	1. Echocardiographic routine variables 2. Electrocardiogram variables 3. Clinical/Haematological variables	ML derived three phenogroups of HFpEF which varied in diastolic dysfunction, hospitalisation, and mortality.
Omar et al., (56)	Diastolic function grading	Random forest, artificial neural network, and support vector machine	1. Echocardiographic strain variables	ML predicted invasively measured PCWP ≥ 18 mmHg with AUC = 0.88. ML correctly identified 80% of patients with raised PCWP, with no indeterminate classifications.
Lancaster et al., (19)	Diastolic function grading, prognostication	Unsupervised hierarchical clustering	1. Echocardiographic routine variables	ML found two distinct clusters in those who would normally be ‘screened' for diastolic dysfunction with the guideline (9); one cluster was mostly (72%) guideline-normal whilst the other cluster was mostly (also 72%) guideline-defined dysfunction or indeterminate grading.
Tokodi et al., (57)	Diastolic function grading, prognostication	Unsupervised topological data analysis and clustering	1. Echocardiographic routine variables 2. Major adverse cardiac event hospitalisation records	Continuous ‘patient similarity networks', derived with ML and later split into segments, vary in diastolic function, mortality, and morbidity, with no indeterminate classifications.
Cho et al., (59)	Diastolic function grading, phenotyping	Unsupervised topological data analysis, supervised decision tree, ensemble and deep neural network	1. Echocardiographic routine diastolic variables 2. Echocardiographic strain variables 3. Vector flow mapping variables	ML produced a patient similarity network with four regions and no indeterminate classifications—regions linearly progressed in terms of diastolic variables, heart failure stages A-D and New York Heart Association functional classes.
Pandey et al., (60)	Diastolic function grading, phenotyping, prognostication	Unsupervised topological data analysis, agglomerative hierarchical clustering and supervised deep neural network	1. Echocardiographic routine diastolic variables	ML was superior to ASE 2016 diastolic guideline grades for predicting invasively-measured elevated left ventricular filling pressure (AUC = 0.88 vs. 0.67). Two clusters of patients were found—the high-risk phenogroup showed higher rates of heart failure hospitalization and/or death than the low-risk phenogroup in multiple external validation cohorts.

*ML, machine-learning; HFpEF, heart failure with preserved ejection fraction; AUC, area under the receiver operator curve; PCWP, pulmonary capillary wedge pressure*.

ML's ability to effectively combine diastolic information to improve disease identification is also demonstrated by Sengupta et al. (43) who used an associative memory classifier–based ML algorithm to differentiate constrictive pericarditis from restrictive cardiomyopathy. The AUC of 0.89 for speckle-tracking strain variables increased to 0.96 when just four routine diastolic pertinent variables were included in their model (septal thickness, posterior wall thickness, e' and E/e').

Further evidence comes from Choi et al. (44) who tested a range of different ML algorithms for their ability to diagnose both HFpEF and systolic HF with a range of clinical, blood, electrocardiographic and echocardiographic variables. The diastolic pertinent variables combined included EF, indexed mass, septal E/e' and tricuspid regurgitation maximum velocity (TR-Vmax). When compared to physician diagnosis, ML diagnosed HFpEF with 99.6% concordance.

Novel parameters of diastolic dysfunction, relevant to breathless patients, hypertensives and those with HFpEF, have been elucidated through ML. Sanchez-Martinez et al. (36) obtained tissue Doppler data during exercise echocardiography, and *via* unsupervised agglomerative hierarchical clustering ML identified premature fusion of early and late diastolic waves, increased variability in the onset of atrial contraction (a′ wave) and a blunted response in atrial velocities (a′ wave peak) during exercise as novel diastolic variables for assessing those with, or at risk of, HFpEF. The importance of these in other populations and their potential clinical utility remains unknown.

### ML for Diastolic Phenotyping and Prognostication

Over the last 5 years or so a rapidly growing body of evidence has accumulated where ML has been applied to phenotype patient's diastology based upon clinical and echocardiographic data. Most often for this purpose, unsupervised ML cluster analysis has been used, which groups patients in a potentially novel way based upon input data similarities. Commonly the disease of interest has been HF, with different authors finding two (45, 46), three (47–49), four (50) and even six (51) phenogroups of HFpEF when applying ML clustering to echocardiographic variables.

For example, Nouraei et al. (51) found six clusters of HFpEF which varied in diastolic dysfunction, endpoints and clinical features. Echocardiographic diastolic variables they used included indexed LA volume, indexed mass, E/A ratio, average E/e', tricuspid regurgitation maximum velocity and grade of diastolic dysfunction. Segar et al. (49) found three phenogroups of HFpEF which varied in diastolic dysfunction, BNP, comorbidities, mortality and hospitalisations. E/A ratio and LA area were among the optimal 20 variables for predicting phenogroup membership.

Hedman et al. (45) found two HFpEF clusters based upon 32 echocardiographic variables, which was possible despite >50% of the patients being in atrial fibrillation at the time of the scan. This supports the notion that ML does not necessarily need individual parameters like E/A ratio all of the time to grade diastolic function, hence offering a more flexible approach than the current clinical methodology.

Among acute HF admissions Horiuchi et al. (52) undertook cluster analysis which yielded three phenogroups that varied in diastolic function, risk of death and subsequent hospitalisation. This highlights the ability of ML to create novel groupings, or classifications, that are not only diagnostically important, but that are also prognostically important. ML can provide clinically useful results in conditions other than HF as shown by Mishra et al. (53), who performed cluster analysis of clinical and echocardiographic data from stable coronary artery disease patients with seven years mean follow-up. Four phenogroups resulted, which varied in diastolic dysfunction and hospitalisation risk.

Unique insights about non-cardiac chronic conditions are also possible with ML of diastolic variables. The relationship between diastolic function and renal function, in the setting of type 2 diabetes, was the subject of investigation by Pecková et al. (54). Unsupervised ML clustering found two subgroups of those with impaired renal function: when there was an early diastolic tissue velocity e' ≤ 7.1 cm/s there was a significant correlation between the echocardiographic ratio E/e' and renal function, whereas when e' > 7.1 cm/s then there was no significant correlation. This highlights the aforementioned potential for ML to unearth associations which may not be obvious to the human eye nor with traditional statistics.

### Grading Diastolic Function With ML

Another application of ML is for grading of diastolic function. Evidence exists suggesting that ML can overcome some of the confounding factor, indeterminate classification and accuracy barriers surrounding the routine clinical guideline grading of diastolic function with echocardiography. Omar et al. (55) investigated whether ML of LV and LA speckle-tracking strain (STS) variables could assess LV diastolic function independently of routine Doppler parameters. They undertook cluster analysis using nine STS variables from 130 patients with heart failure symptoms. This produced three clusters which varied concordantly in diastolic Doppler indices and LA maximal volume, with no indeterminate classifications. The clusters were invasively validated in a further 44 patients, where PCWP and LV-EDP increased concordantly across their three pre-identified clusters.

These findings show that ML can identify discrete phenotypes of diastolic function which vary in severity, much like the current grading system, but which do not rely upon the acquisition of standard diastolic variables. This could greatly assist healthcare professionals in identifying those with diastolic dysfunction when technical limitations or missing Doppler variables may preclude following the guideline grading algorithms. How these novel ML results relate to clinical markers of diastolic dysfunction and heart failure, like symptoms and b-type natriuretic peptides, is unclear.

In an extension of their work, Omar et al. published further analysis from the same patient cohort (56). Using 14 STS parameters they trained three separate ML algorithms (random forest, artificial neural network, and support vector machine) in a supervised fashion to diagnose raised filling-pressures, with E/e' or PCWP as a label and a majority voting system to decide the outcome. Taking the best 11 STS parameters, an AUC = 0.85 was obtained for predicting E/e' ≥ 13 in the derivation group, with AUC = 0.88 for predicting PCWP ≥ 18 mmHg in the invasive validation group. ML of the echocardiographic parameters correctly identified 80% of patients with raised PCWP, with no indeterminate classifications, exceeding the performance of the guideline grading algorithm in most invasive studies of its accuracy (as per section Clinical guideline accuracy). A limitation of this work is that referral for heart failure symptoms created a biased population in terms of diastolic function.

Lancaster et al. (19) analysed routine diastolic parameters in a retrospective analysis of 866 consecutive patients referred for myocardial function assessment. Scans were grouped according to which ASE/EACVI guideline algorithm (9) would be applied clinically: screening or grading. Unsupervised ML cluster analysis in the screening group found two clusters with no indeterminate classifications. The first larger cluster (*n* = 460, 82% of 559) contained mostly guideline classified “normal” diastolic function (72%), whilst the second smaller cluster (*n* = 99, 18%) contained mostly guideline classified diastolic dysfunction or “indeterminate” (72% of 99). Agreement between ML and guideline classification of diastolic dysfunction was poor (kappa = 0.41).

In the grading group, ML again found two clusters: one (*n* = 236, 61%) comprised mainly guideline graded “mild” (44%) and “moderate” (50%), with little “severe” (6%). In contrast, the second (*n* = 151, 39%) contained mostly “moderate” (78%) with some “severe” (15%) and infrequently “mild” (7%). Using binary classification of mild vs. moderate/severe, the agreement between ML and guideline grading was better than for screening (kappa = 0.62). Given the known guideline limitations, a modest agreement metric should not discourage the notion of a more effective ML grading system.

This evidence leads to some interesting questions. There was a lot of overlap between their two clusters for those who the guideline grades as having moderate diastolic dysfunction. Does the traditional “moderate” category perhaps contain two phenotypes, one with normal filling-pressures and one with raised? Furthermore, given the convention of associating moderate/severe with raised filling-pressures, is binary grading (normal/raised filling-pressures) clinically more useful than the historic four grades (normal/mild/moderate/severe)?

Additionally, the authors found their ML clusters to show improved prognostication compared to the ASE/EACVI guideline. Their ML diastolic dysfunction screening algorithm better predicted event-free survival and rehospitalisations whilst their grading algorithm was better at predicting mortality but was indifferent to the guideline for rehospitalisations. Again, this hints that a dichotomous classification system may better correspond to patient outcomes.

Placing patients onto a continuous spectrum from normality to disease, rather than aggregated into categorical boxes, represents an achievable aspiration for ML and would facilitate a major leap towards personalised medicine for diastology. Tokodi et al. (57) applied “Topological Data Analysis” and unsupervised clustering ML to the same cohort of 866 patients used by Lancaster et al. (19). The TDA technique is well-suited for detecting subtle geometric patterns in high dimensional data (57, 58). The authors studied nine echocardiographic parameters used for diastolic classification (EF, indexed LV mass, E, A, E/A, e', E/e', LA indexed volume and TR velocity). Their initial analyses created a “patient similarity network” (57) which cluster analysis divided into four regions. These varied in cardiac structure, LV systolic/diastolic function, mortality, morbidity, and diastolic dysfunction risk-factors, with no indeterminate classifications.

The authors also tested the prognostic ability of their loop in *n* = 96 completely new patients with two serial echocardiograms each. A supervised random-forest ML algorithm was trained with the derivation cohort data with loop region as a label. In the unseen validation cohort, the ML loop region was associated with major adverse cardiac event hospitalisation (MACE-h). Upon comparing the first and second echocardiograms, an improvement to (or remaining within) a lower risk region was associated with lower MACE-h rates. These results support the notion that ML can identify patients with different stages of diastolic dysfunction in a personalised fashion, with fidelity for linking diastolic changes to outcomes. How their loop-regions correspond to guideline diastolic grades, and to invasive filling-pressures, would be of interest.

Whilst the other pieces of evidence concerning ML classification of diastolic dysfunction have used either STS or routine Doppler diastolic parameters exclusively, added benefit may be realised by simultaneously applying ML to novel variables emerging from the research setting. Cho et al. (59) prospectively recruited *n* = 247 consecutive patients and *n* = 50 healthy control participants. All routine parameters needed to perform guideline classification of LV diastolic function were obtained, apart from lateral e'. LV and LA deformation, plus vector flow mapping (VFM) parameters were also measured.

Topological Data Analysis produced a patient similarity network with four regions and no indeterminate classifications—regions linearly progressed in terms of diastolic parameters, heart failure stages A-D and New York Heart Association functional classes I-IV. Three supervised ML techniques were then individually trained with the label of network region. A Deepnet neural network performed best at classifying scans into regions when given all 42 variables (AUC between 0.83-0.99 for the four regions). Of the 25 most important variables, 44% were VFM, 40% routine and only 16% STS. A high dependency of the model upon niche VFM variables, which most cardiologists and echocardiographers are not skilled in measuring, perhaps limits the potential for adoption of these findings into current clinical practice. Interestingly and reassuringly in this study though, the regions of the patient similarity network appeared to mimic the four-grade system of the guidelines.

Pandey et al. (60) are the first to have applied deep learning to the assessment of diastolic function from echocardiography data. As in the work of Tokodi et al. (57) and Cho et al. (59), a patient similarity network was firstly derived with Topological Data Analysis of the routine parameters needed to follow guideline diastolic assessment. Two network regions were defined with the help of clustering; high-risk and low-risk for heart-failure hospitalisation or cardiac death. Supervised deep learning with a neural network model then classified phenogroup membership before further evaluations were undertaken in multiple independent datasets with invasive haemodynamic, outcome, cardiac biomarker, and exercise performance metrics.

The model of Pandey et al. (60) showed better prediction than the guideline diastolic grades for elevated LV filling-pressure > 15 mmHg (AUC = 0.88 vs. 0.67; *p* < 0.01). Tellingly, most of the outperformance was driven by guideline “indeterminate” subjects, as when removed the AUC were similar between the deep-learning model and guideline method. Furthermore, the high-risk phenogroup showed higher rates of heart failure hospitalisation and/or death than the low-risk group in multiple HFpEF trial datasets. As such, these results further support a clinically meaningful augmentation of diastolic assessment with ML of echocardiographic variables.

### Requisites for ML Translation to Clinical Practice

**Figure 2:**
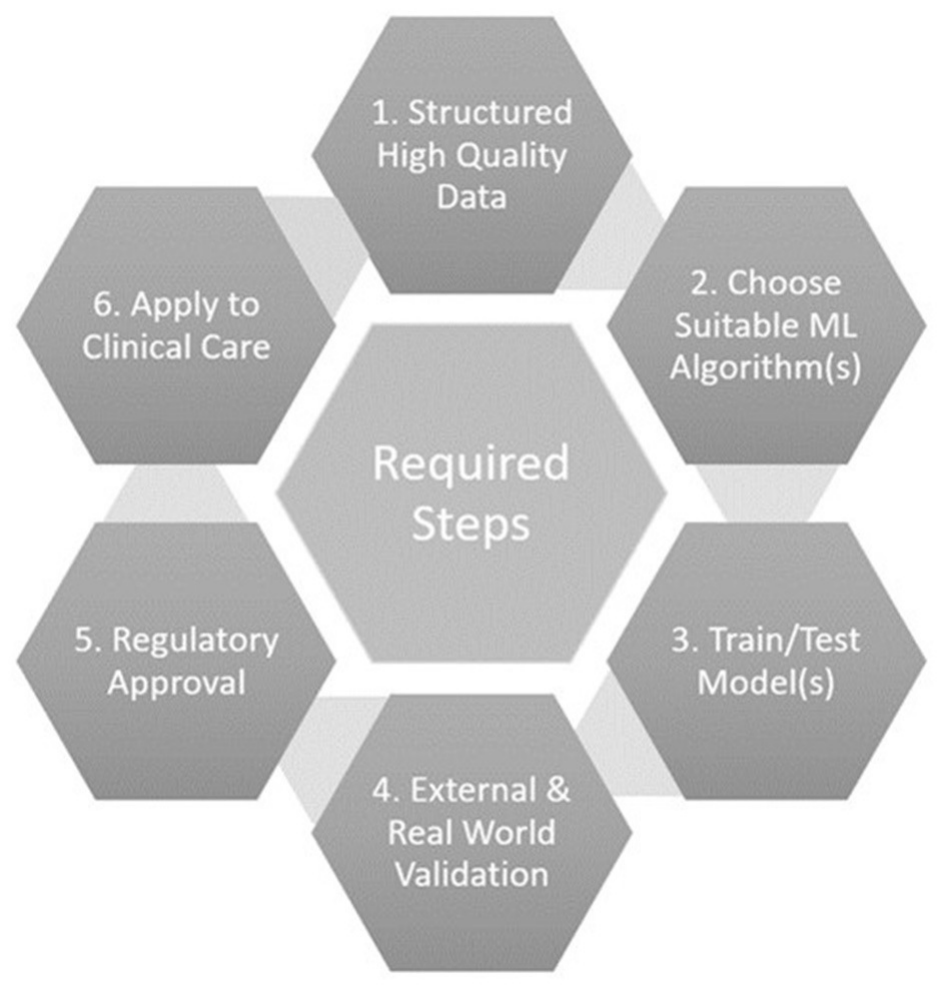
Key steps required for using machine-learning for echocardiographic diastolic analysis.

Several important steps are required to effectively use ML for echocardiographic diastolic function analysis, and subsequently translate the results into clinical practice (Figure 2). Firstly, high quality structured datasets, with sufficient granularity to describe the system of interest, are required. These are traditionally obtained through clinical trials, however, clinical archives are now often mined due to the lower resource costs and need for large quantities of training data that reflects real life practice (38). Care must be taken to ensure that the raw data is still acquired following high-quality protocols, is in a suitable format for ML ingestion (e.g., normalised, standardised), and consideration of missing data and outliers is paramount.

Secondly, a choice of suitable ML algorithm(s) to train should be made by considering the research aims. For classification, where labelled data is available and the so called “ground-truth” is known, a supervised algorithm such as a support vector machine or decision tree would be considered. If the aim is to derive novel groupings or phenotypes, and the data is unlabelled, then unsupervised methods such as clustering algorithms or principal component analysis are better suited. Some aims may require several algorithms in combination, i.e., an ensemble.

Training of the ML model with the data, tuning of model hyperparameters, and performance testing form the next stage of the process. Validation with independent external datasets is recommended to assess performance of the model, however investigators often do not have access to such data and hence frequently use cross-fold validation. This entails splitting data into sections (known as folds), using some folds to train and some folds to test the model, before rotating the folds around the whole dataset (61). Traditional statistical analysis methodologies are normally applied to the resulting outputs where, for example, well-known metrics such as sensitivity, specificity, and accuracy, allow appreciation of performance and a comparison of models for a classification task. These hence permit identification of the optimal model or ensemble.

Variations in equipment, protocols, staff, and patients between datasets may influence the performance of a model. Validation with independent external datasets hence allows appreciation of the real-world applicability or generalisability of the findings, which is especially important if the training dataset was from a clinical trial. Overfitting, where the performance of the model is high in the training dataset but lower in other data, suggests that the model has leant the training data well but lacks generalisability. Underfitting can be found where the training data was too small and/or of too low a dimensionality to allow the model to accurately perform the desired task. Validation thus helps to check for over/under-fitting of the ML model, which is particularly relevant if the data came from a specific cohort, demographic, or location for example.

Once a ML algorithm has been trained, tested, and validated, and there is confidence that the model can positively impact clinical care, regulatory approval should be sought to enable widespread adoption or commercialisation. Regulatory approval by the Food and Drug Administration in America, or with the Conformité Européene (CE) mark in Europe has been increasingly sought in recent years for devices incorporating ML (62). Given the potential impact upon patient care, ML algorithms should be certified as a medical device to make sure that they are safe and fit for purpose, and to provide reassurance of quality to purchasers, users, and patients. The risk category being assessed under by the regulatory body, and transparency of the submitted information, are the subject of debate (63).

The final step of introducing a produced ML tool into clinical care then requires some more considerations. Regular audit must be undertaken to ensure safety and effectiveness of decisions made as a result of the tool introduction. The outcome data produced by the ML tool, such as a predicted diagnosis, should be regularly compared to a reference standard or clinical diagnosis. The ML output(s) could then feedback into the entire development cycle of another ML tool, because of this potential alteration in patient care.

## Potential Benefits, Challenges, and Future Directions

With the applications of ML augmented echocardiography now stepping out of the shadows for diastolic assessment, a number of potential benefits and challenges can be seen (Table 2). From the healthcare system perspective, improved prognostication of cardiology patients with ML may facilitate efficient resource allocation, meaning that the right care is available to the right patient at the right time.

**Table 2:** Challenges and potential benefits of machine-learning augmented echocardiography for diastolic analysis.

**Machine-Learning Augmented Echocardiography for Diastolic Analysis**	
**Potential benefits**	**Challenges**
•Improved diagnostic accuracy •Enhanced prognostication •Reduced indeterminate classifications •Assessment in presence of confounding factors •Personalised diastolic assessment •Use all scan information collected •Discover novel diastolic parameters •Automated assessment •Track serial changes	•Lack of echocardiographic data with simultaneous invasive haemodynamics •Quality of clinical datasets for training •Quantity of records in training datasets •To be robust to changes in pre-load / after-load •“Black-box” perception •Regulatory approval •How to best integrate into clinical practice?

In the clinical echocardiographic laboratory, healthcare staff may benefit from automated assessment tools which could save them time, reduce inter- and intra-rater variability and allow for subtle serial changes to be monitored. ML may be able to negate the influence of confounding factors and reduce the impact of a missing routine diastolic parameter, creating a more robust and widely applicable technique. Use could also be made of all the rich detail collected in an echocardiogram, which alongside detection of latent data relationships and novel parameters could vastly improve diagnostic accuracy for diastolic dysfunction. All of these aspects are likely to be the focus of future research.

From the perspective of the physician, a personalised diastolic assessment with ML would allow tailored investigations and patient management. A reduction or complete elimination of indeterminate diastolic grading would reduce clinical uncertainty and lessen the need for additional complex or invasive investigations, such as cardiac catheterisation. This would also facilitate better decision making in high-risk groups where diastolic status may alter risk-benefit balances of interventions.

Several challenges are apparent though for ML augmented diastolic assessment. Firstly, the thirst of ML algorithms for high-quality, large-volume, high-dimensionality training data is a problem in a world where healthcare systems are still struggling to digitalise and integrate electronic systems. Significant progress has been achieved thus far with modest sample sizes, but a truly “big-data” approach to diastolic assessment seems warranted to validate existing findings, to unlock new insights, and to increase the clinical applicability of results. Limitations of the current evidence are that often patients with missing data, or indeterminate guideline classification, are excluded from studies. Where these patients fit on the spectrum of diastology would be of great interest given their prevalence.

Secondly, a lack of direct invasively measured diastolic data, to use as a “ground truth,” also limits ML research about diastolic function in many populations, as patients simply do not receive such a test routinely. Given ethical and resource considerations, it is unlikely that this can be overcome. A third challenge is that a ML diastolic assessment tool would require resilience to changes in preload, afterload, and heart rate, etc. Given oscillatory patterns of, for example, de-compensated heart failure and subsequent treatment, the field should aim to not only categorise diastolic dysfunction, but also to assign a personalised “live” diastolic status, which could then be used to track temporal changes in diastolic function. This would greatly advance the field towards precise and personalised medicine.

Fourthly, a perception that ML represents a “black box” technology, where healthcare professionals do not understand how it is arriving at a decision, is also a problem for ML augmented echocardiography. Input parameter feature weightings, and heat maps of image areas being used by the machine, offer ways for researchers to dispel this perception. Finally, regulatory approval also acts as a potential obstacle for ML innovators.

The field of echocardiography is moving towards a more automated, data-driven, and analytical approach. Diastolic function is not something that can be readily eyeballed—it needs expert clinical insight to meet rigorous science to improve its assessment. How to best integrate a ML diastolic assessment tool into clinical practice should form the basis of future debate.

## Conclusions

Evidence shows that ML can use diastolic parameters to differentiate diseases, improve the accuracy of disease diagnoses, and identify diastolic phenotypes within heterogeneous conditions such as HFpEF. There is also evidence to suggest that ML can improve identification of raised filling-pressures, classify or grade diastolic function in novel ways, and improve upon the prognostic ability of the current diastolic clinical standard. Although there are numerous potential benefits, many challenges stand in the way of progress for the field. ML augmented echocardiography for diastolic assessment is here, but real-world applicability and its relationship to clinical decision making remains to be seen.

## Author Contributions

AF and PL conceptualised the review topic. AF performed the literature search and drafted the article. All authors edited and reviewed the article and approved the manuscript for submission.

## Conflict of Interest

PL acknowledges current grant support related to medical imaging from the British Heart Foundation, Wellcome Trust, National Institute of Health Research and Lantheus Medical Imaging. PL is a stockholder, non-executive director and co-founder of Ultromics: a medical imaging artificial intelligence company. The remaining authors declare that the research was conducted in the absence of any commercial or financial relationships that could be construed as a potential conflict of interest.

## Publisher's Note

All claims expressed in this article are solely those of the authors and do not necessarily represent those of their affiliated organizations, or those of the publisher, the editors and the reviewers. Any product that may be evaluated in this article, or claim that may be made by its manufacturer, is not guaranteed or endorsed by the publisher.
